# Physical, Functional and Genetic Interactions between the BEACH Domain Protein SPIRRIG and LIP5 and SKD1 and Its Role in Endosomal Trafficking to the Vacuole in Arabidopsis

**DOI:** 10.3389/fpls.2017.01969

**Published:** 2017-11-20

**Authors:** Alexandra Steffens, Marc Jakoby, Martin Hülskamp

**Affiliations:** Botanical Institute, Cologne Biocenter, University of Cologne, Cologne, Germany

**Keywords:** SPIRRIG, BEACH domain containing protein, Arabidopsis, endosomes, vacuolar transport

## Abstract

Beige and Chediak Higashi (BEACH) domain-containing proteins (BDCPs) are facilitators of membrane-dependent cellular processes in eukaryotes. Mutations in BDCPs cause malfunctions of endosomal compartments in various cell types. Recently, the molecular analysis of the BDCP homolog gene *SPIRRIG* (*SPI*) has revealed a molecular function in P-bodies and the regulation of RNA stability. We therefore aimed to analyze, whether SPI has also a role in membrane-dependent processes. In this study, we show that SPI physically interacts with endosomal sorting complex required for transport associated ATPase Suppressor of K^+^-transport growth defect1 (SKD1) and its positive regulator, LYST Interacting Protein 5 (LIP5) and report genetic interactions between *SPI* and *SKD1* and *LIP5*. We further show that the endosomal transport route of soluble proteins to the lytic vacuole is disturbed in *spi lip5* double mutants but not in the single mutants. These vacuolar transport defects were suppressed by additional expression of SKD1. Our results indicate that the BEACH domain protein SPI has in addition to a role in P-bodies a function in endosomal transport routes.

## Introduction

Beige and Chediak Higashi (BEACH) domain-containing proteins (BDCPs) were first identified during the characterization of the human Chediak Higashi Syndrome (CHS) and the *Beige* phenotype in mouse (Barbosa et al., 1996; Perou et al., 1996). CHS is a lethal autosomal recessive disorder, hallmarked by complex clinical manifestations comprising bleeding diathesis, albinism, immunodeficiency, and neurodegeneration (Introne et al., 1993; Kaplan et al., 2008). At the cellular level, CHS patients show abnormally enlarged lysosomes and protein sorting and secretion defects in various cell types (Brandt et al., 1975; Zhao et al., 1994; Barbosa et al., 1996; Perou et al., 1996; Spritz, 1998; Introne et al., 1999; Ward et al., 2000). The corresponding gene was therefore termed *Lysosomal Trafficking Regulator* (*LYST*) (Barbosa et al., 1996; Nagle et al., 1996; Perou et al., 1996).

The LYST protein contains three conserved motifs at the C-terminus: a Pleckstrin-Homology Domain (PH-Domain), the BEACH domain and a WD40 repeat motif. WD40 repeats and PH-Domains are known to mediate heterotypic protein interactions and protein recruitments to membranes via phospholipid binding, respectively (Neer et al., 1994; Lemmon, 2007). The molecular function of BEACH domains is not known. BDCPs, exhibiting a protein domain organization like LYST, are present in all eukaryotic species and mutants have been isolated in various organisms. Some of the most prominent examples are *Neurobeachin* (*Nbea*) deficient mice, characterized by impaired retrieval and post-Golgi trafficking of neuronal neurotransmitter receptors (Wang et al., 2000; del Pino et al., 2011); Drosophila *Blue Cheese* (*bchs*) mutants in which Ras related in brain protein 11 (RAB11)-dependent vesicle trafficking events at the Trans-Golgi Network (TGN), Golgi-derived vesicles and recycling endosomes are disturbed (Khodosh et al., 2006); *C. elegans* Suppressor/Enhancer of Lin-12 Protein 2 (SEL2) mutants that exhibit protein sorting defects between the cell surface and lysosomes (de Souza et al., 2007); Dictyostelium deficient for LvsA or LvsB (Large Volume Sphere A and B), showing hyper-accumulations of the vacuolar H^+^-ATPase in early endo- and phagocytotic compartments (Gerald et al., 2002; Wu et al., 2004) and enhanced lysosomal enzyme secretion under starvation (Harris et al., 2002); and *Arabidopsis thaliana bchD* (*BEACH domain protein D*, also termed *green fluorescent seed 12*) mutants, in which protein sorting to the plant specific protein storage vacuoles (PSVs) in seeds, but not in vacuolated vegetative tissues, is compromised (Teh et al., 2015). Because of the commonalities between these phenotypes, BDCPs are considered to be involved in the regulation of endosomal sorting processes (Cullinane et al., 2013).

In plants, the SPIRRIG gene in *Arabidopsis thaliana* is the best-studied BDCP representative [SPI, also termed BEACH domain protein A1 (BchA1) (Teh et al., 2015)]. SPI deficient plants share all morphological phenotypes observed in Arabidopsis mutants with defects in the ARP2/3- (actin related proteins 2 and 3) and SCAR/WAVE- (suppressor of cAMP receptor from Dictyostelium/Wiskott Aldrich syndrome protein family verprolin-homologous protein) complex mediated actin filament polymerization and branching (Hulskamp et al., 1994; Szymanski et al., 1999; Saedler et al., 2009). These include twisted and wavy trichomes, reduced length of root hairs, cell attachment defects and less complex epidermal pavement cells (Saedler et al., 2009). In contrast to all other mutants of this class, *spi* mutant cells do not show any defect in actin cytoskeleton organization (Schwab et al., 2003). In addition, SPI is involved in salt stress response (Steffens et al., 2015). In this context the analysis of its molecular function revealed a role in post-transcriptional stabilization of mRNAs (Steffens et al., 2015). This raises the question, whether the BDCP SPI gene has a different molecular function as reported for other BDCPs or whether SPI has a dual role in post-transcriptional regulation of mRNA and endomembrane dynamics. A possible role of the Arabidopsis SPI protein in endomembrane dynamics was initially suggested by the finding that root hairs show fragmented vacuoles (Saedler et al., 2009). Plant vacuoles are thought to originate from the endoplasmic reticulum (ER) as part of the secretory pathway (Matile, 1968; Mesquita, 1969; Viotti et al., 2013; Zhang et al., 2014). As a consequence, vacuoles receive cargo molecules from both, the anterograde transport route from the ER and the retrograde trafficking pathway from the plasma membrane (Saint-Jore-Dupas et al., 2004; Scheuring et al., 2011). The latter pathway involves the evolutionarily highly conserved ESCRT (endosomal sorting complex required for transport) machinery that is essential for the recognition of ubiquitinated cargo molecules destined for the vacuolar/lysosomal decay at membranes of maturating endosomes (Hurley and Emr, 2006; Spitzer et al., 2009; Richardson et al., 2011; Scheuring et al., 2012; Gao et al., 2015). Following the deubiquitination of cargo molecules (Katsiarimpa et al., 2014), the AAA^+^-type ATPase SKD1 (Suppressor of K^+^-transport growth defect 1/also termed VPS4; Vacuolar Protein Sorting-associated protein 4) triggers the final steps of endosome maturation to multivesicular bodies (MVBs), the fission of intraluminal vesicles (ILVs) and dissociation of ESCRT components from the endosomal membrane (Babst et al., 1998; Sachse et al., 2004; Babst, 2005; Lata et al., 2008; Landsberg et al., 2009). The expression of dominant negative SKD1 causes the formation of abnormally large MVBs containing a reduced number of ILVs concomitant with a reduced vacuolar transport and secretion of intraluminal cargo (Raymond et al., 1992; Fujita et al., 2003; Haas et al., 2007; Shahriari et al., 2010b; Scheuring et al., 2012). A possible function of BDCPs in the ESCRT pathway is suggested by the finding that human LYST interacting protein 5 (LIP5) interacts with the LYST in yeast two-hybrid assays (Tchernev et al., 2002). LIP5 is a positive regulator of SKD1 in mammals, yeast, and plants (Fujita et al., 2004; Azmi et al., 2006; Haas et al., 2007). The expression of dominant negative SKD1 enhances the membrane association of LYST in cultured human cells (Fujita et al., 2004) suggesting that the interaction of LIP5 and LYST is functionally relevant.

In this study, we assessed the role of the BDCP SPI protein in the ESCRT regulatory pathway. We demonstrate that SPI physically interacts with LIP5 and SKD1 from *Arabidopsis thaliana*. By combining molecular and biochemical approaches, we demonstrate that SPI is involved in the regulation of SKD1 and LIP5 and the route of endomembrane trafficking.

## Results

### The Arabidopsis BDCP SPI Protein Interacts with LIP5 and SKD1

**FIGURE 1:**
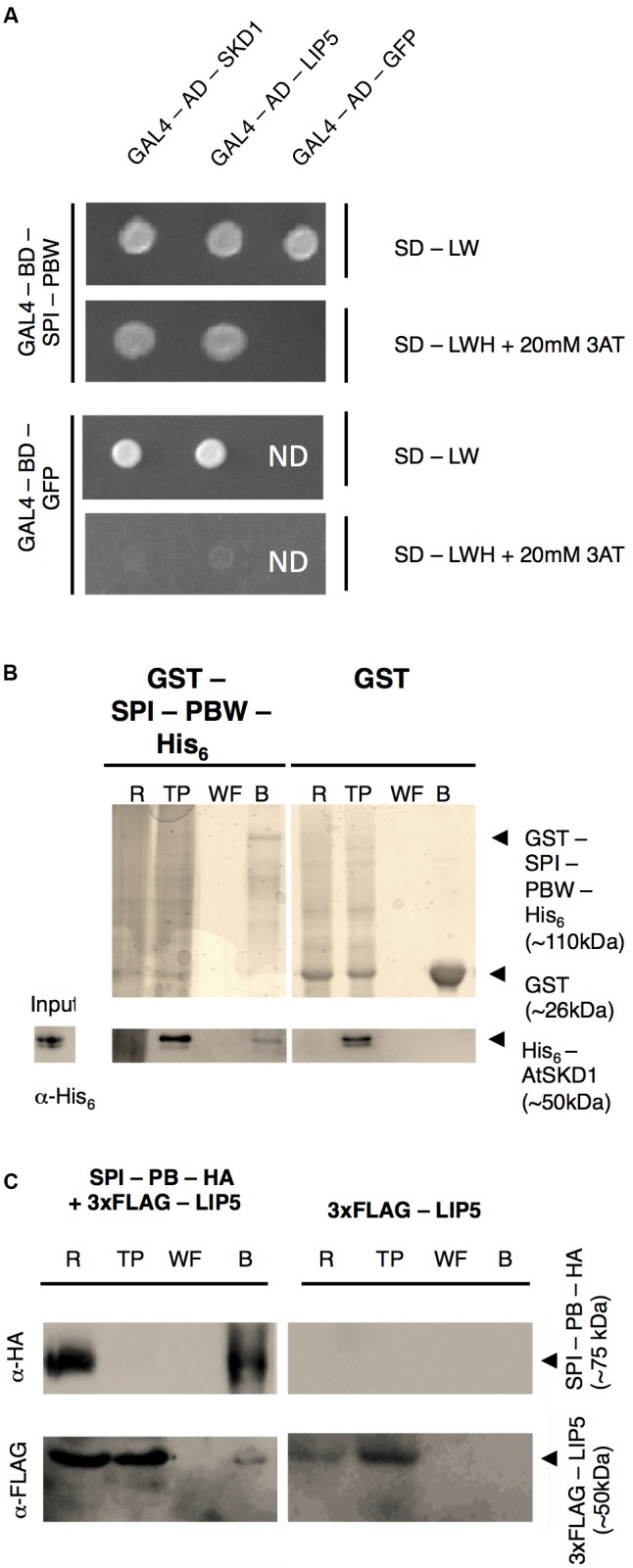
SPI interacts with SKD1 and LIP5. **(A)** Yeast two-hybrid interactions between SPI-PBW, SKD1, and LIP5. Top: double transformed yeast cells on dropout medium lacking leucine (-L) and tryptophan (-W). Bottom: interaction between GAL4-Binding Domain (BD) fusions of SPI-PBW, and SKD1 or LIP5, N-terminally fused to the GAL4 Activation Domain (AD), on dropout medium lacking leucine (-L), tryptophan (-W) and histidine (-H), supplemented with 20 mM 3-Aminotrizole (3AT). GAL4-AD-GFP (Green Fluorescent Protein) and GAL4-BD-GFP vectors served as negative controls. ND, not determined. **(B)** Co-precipitation of bacterially expressed SPI-PBW and SKD1. Top: Purifications of GST-SPI-PBW-His_6_ (∼110 kDa) and the negative control GST (∼26 kDa) are shown on Coomassie stained gels. Bottom: Input of purified His_6_-SKD1 (∼50 kDa) and co-precipitations of His_6_-SKD1/GST-SPI-PBW-His_6_ detected by α-His_6_ antibody staining. No co-precipitation was observed between GST and His_6_-SKD1. Expected protein sizes are indicated by arrowheads. R, raw extract; TP, throughput; WF, last wash fraction; B, beads fraction. **(C)** Co-immunoprecipitation of SPI-PB-HA and 3xFLAG-LIP5 from lysates of transfected *N. benthamiana* leaves. Top: Immunoprecipitation of SPI-PB-HA (∼75 kDa) was detected by α-HA antibody staining on a Western blot. Bottom: 3xFLAG-tagged LIP5 (∼50 kDa) was detected in R, TP and the B of SPI-PB-HA co-transfected leaves by α-FLAG antibody staining on Western blots. 3xFLAG-tagged LIP5 alone was not precipitated with α-HA-beads. Expected protein sizes are indicated by arrowheads. ProteinA beads were included as a negative control for all proteins tested (Supplementary Figure S1B).

In a first step, we examined the protein interactions of the Arabidopsis BDCP SPI with LIP5 and SKD1. As large size of the SPI cDNA renders the molecular analysis extremely difficult, we used the evolutionarily conserved C-terminal part containing the PH-BEACH-WD40 domains (called SPI-PBW hereafter, Supplementary Figure S1A) for protein-interaction assays. Yeast two-hybrid assays revealed interactions between SPI-PBW and both, LIP5 and SKD1 (**Figure 1A**). The interaction between SPI-PBW and SKD1 was confirmed by co-precipitations (co-Ps) of bacterially expressed Glutathione-S-Transferase (GST)/His_6_-fusions of SPI-PBW and His_6_-fusions of SKD1 (**Figure 1B**). His_6_-SKD1 did not bind to GST labeled resins, confirming the specificity of the SPI-PBW/SKD1 interaction. As the bacterial expression of His_6_-LIP5 failed, we confirmed the SPI/LIP5 interaction in co-immunoprecipitation (co-IP) experiments with proteins expressed in *Nicotiana benthamiana* (*N. benthamiana*) leaf cells. Here, the expression of SPI-PBW was very weak and just above the detection level. We therefore used a slightly smaller SPI fragment lacking the WD40-repeats (termed SPI-PB; Supplementary Figure S1A). SPI-PB was C-terminally fused to a HA-tag (SPI-PB-HA). LIP5 was used as N-terminal fusion with three FLAG tags (3xFLAG-LIP5). These experiments revealed a clear co-IP suggesting an interaction between SPI-PB and LIP5. 3xFLAG-LIP5 did not bind to unlabeled beads, indicating that the SPI-PB/LIP5 interaction is specific (**Figure 1C**). The specificity of the immunoprecipitation was confirmed, by showing that SPI-PB-HA and 3xFLAG-LIP5 did not bind to α-ProteinA beads (Supplementary Figure S1B).

### Intracellular Localization of SPI and Its Interactions with LIP5 and SKD1 at Endosomes

**FIGURE 2:**
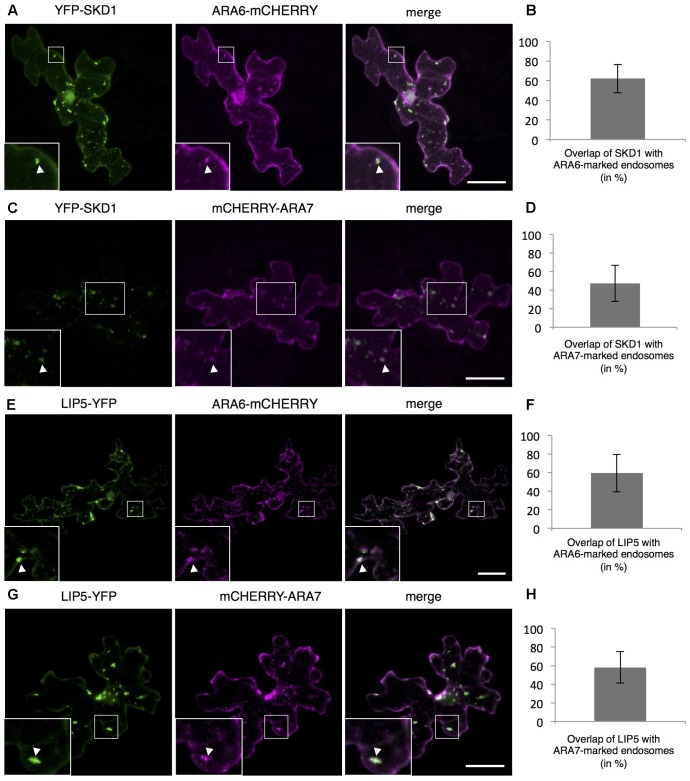
Localization of SPI, LIP5, and SKD1 in plant cells. Representative images of SKD1 or LIP5 co-transformed with endosomal marker proteins and the quantification of co-localization by Manders. **(A)** YFP-SKD1, ARA6-mCHERRY and their overlay. **(B)** Manders coefficient (in %) between YFP-SKD1 and ARA6-mCHERRY. **(C)** YFP-SKD1, mCHERRY-ARA7 and their overlay. **(D)** Manders coefficient (in %) between YFP-SKD1 and mCHERRY-ARA7. **(E)** LIP5-YFP, ARA6-mCHERRY and their overlay. **(F)** Manders coefficient (in %) between LIP5-YFP and ARA6-mCHERRY. **(G)** LIP5-YFP, mCHERRY-ARA7 and their overlay. **(H)** Manders coefficient (in %) between LIP5-YFP and mCHERRY-ARA7. Data denote the average of 20 cells and error bars the corresponding standard deviations (SDs). Scale bars represent 25 μm.

To monitor the intracellular localization of SPI, LIP5 and SKD1, we transiently expressed fluorescently marked proteins. As shown before, we observed YFP-SDK1 either evenly distributed in the cytoplasm or at one or several cytoplasmic dots in transiently transformed Arabidopsis leaf epidermal cells (Supplementary Figures S2A,B) (Shahriari et al., 2010b). Quantification of YFP-SKD1 localization revealed its localization to cytoplasmic dots in about one thirds of the cells (Supplementary Figure S2C). The YFP-SKD1 dots partially co-localized with two endosomal marker proteins, the Rab5 related small GTPases ARA6 (RabF1) and ARA7 (RabF2B) (Ueda et al., 2004; Ebine et al., 2011). Sixty-two percentage of YFP-SKD1 positive dots were labeled with ARA6-mCHERRY and 47.4% were marked with mCHERRY-ARA7 (**Figures 2A–D**). As reported before, LIP5-YFP localized to endosomes (Brandt et al., 1975; Shim et al., 2008; van Balkom et al., 2009). 59.3% of LIP5-YFP labeled dots showed also a ARA6-mCHERRY signal and 58.2% co-localized with mCHERRY-ARA7 (**Figures 2E–H**). In contrast to SKD1 and LIP5, the N-terminal YFP-fusions of SPI-PBW and full length genomic SPI (genSPI) were evenly distributed in the cytoplasm (Supplementary Figures S2D,E).

**FIGURE 3:**
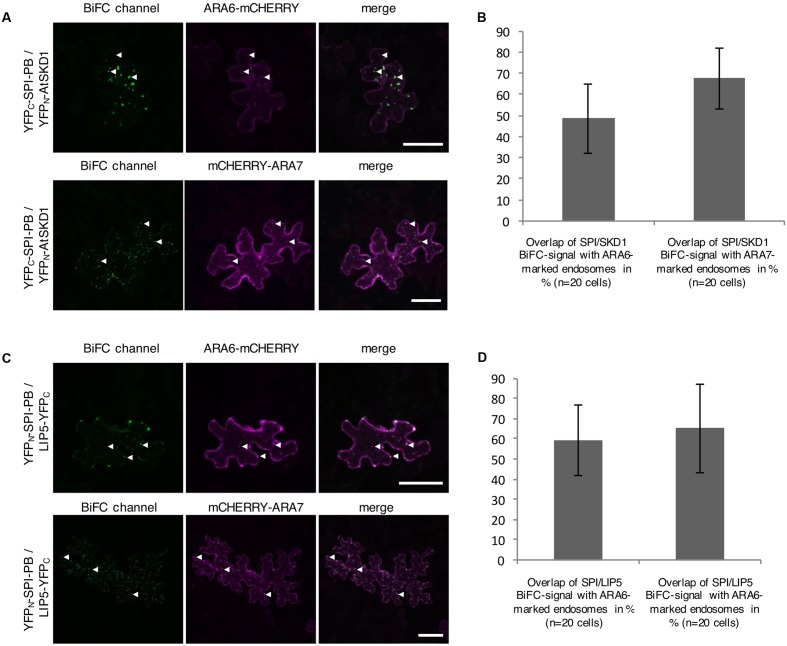
Bimolecular fluorescence complementation (BiFC) interactions of SPI-PBW on endosomes in infiltrated *N. benthamiana* leaf epidermis cells (related to Supplementary Figure S2). **(A)** BiFC interactions of YFP_C_-SPI-PBW and YFP_N_-SKD1 in cells co-expressing ARA6-mCHERRY (Upper) or mCHERRY-ARA7 (Lower). Column III shows the merged pictures of columns I and II. Scale bars represent 50 μm. **(B)** Overlap (in %) between SPI/SKD1 BiFC signals and ARA6- or ARA7-labeled endosomes. Data denote the average of 10 cells. Error bars represent SDs. **(C)** BiFC interaction of YFP_N_-SPI-PBW and LIP5-YFP_C_ in cells co-expressing ARA6-mCHERRY (Upper) or mCHERRY-ARA7 (Lower). Column III shows the merged pictures of columns I and II. Scale bars represent 50 μm. **(D)** Overlap (in %) of SPI-PBW/LIP5 BiFC signals overlapping ARA6- and ARA7-labeled endosomes. Data denote the average of 15 and 12 cells, respectively. Error bars represent SDs.

The intracellular localization of the interaction between SPI-PBW, LIP5, and SKD1 was assessed by Bimolecular fluorescence complementation (BiFC) assays in transfected *N. benthamiana* leaf epidermis cells. We found BiFC interactions of SPI-PBW with LIP5 and SKD1 in cytoplasmic dots. The Manders co-efficient of the SPI-PBW/SKD1 BiFC signal was 54.96% with ARA6-mCHERRY and 45.76% with mCHERRY-ARA7 (**Figures 3A,B**). 49.3% of the SPI-PBW/LIP5 BiFC signal co-localized with ARA6-mCHERRY and 44.6% with mCHERRY-ARA7 (**Figures 3C,D**). As SPI protein fusions are ubiquitously distributed in the cytoplasm we used also a cytoplasmically localized negative control. We found no interactions of SPI-PBW and LIP5 with AtMYC1 (MYC related protein1) (Pesch et al., 2013) (Supplementary Figure S2F). The functional integrity of AtMYC1 N-terminally fused to YFP-fragments was confirmed by demonstrating its BiFC interaction with TTG1 (TRANSPARENT TESTA GLABRA1) (Zimmermann et al., 2004; Zhao et al., 2012) (Supplementary Figure S2G). TTG1-YFP_C_ was included as negative control for YFP_N_-SKD1 (Supplementary Figure S2F). In summary, our data suggest that the interaction between SPI-PBW and LIP5 and SKD1 can occur at endosomes.

Because SPI contains a PH domain known to mediate binding to phospholipids (Ferguson et al., 1995; Lemmon, 2007) we assessed the binding-ability of bacterially expressed GST-SPI-PBW-His_6_ to a selection of membrane-anchored lipids in protein-lipid overlay assays (Supplementary Figures S3A–C). GST was included as a negative control and the PH domain from PLC-d1 as a positive control (Supplementary Figure S3B). We found no binding of GST-SPI-PBW-His_6_ to any of the phospholipids tested, including the endosomal enriched Phosphatidylinositol-3-phosphate (PI3P) (Voigt et al., 2005; Vermeer et al., 2006) (Supplementary Figure S3C). These data suggest that the association of SPI to endosomes is not mediated by phosphoinositides.

### Genetic Analysis of SPI, LIP5, and SKD1 in Arabidopsis

**FIGURE 4:**
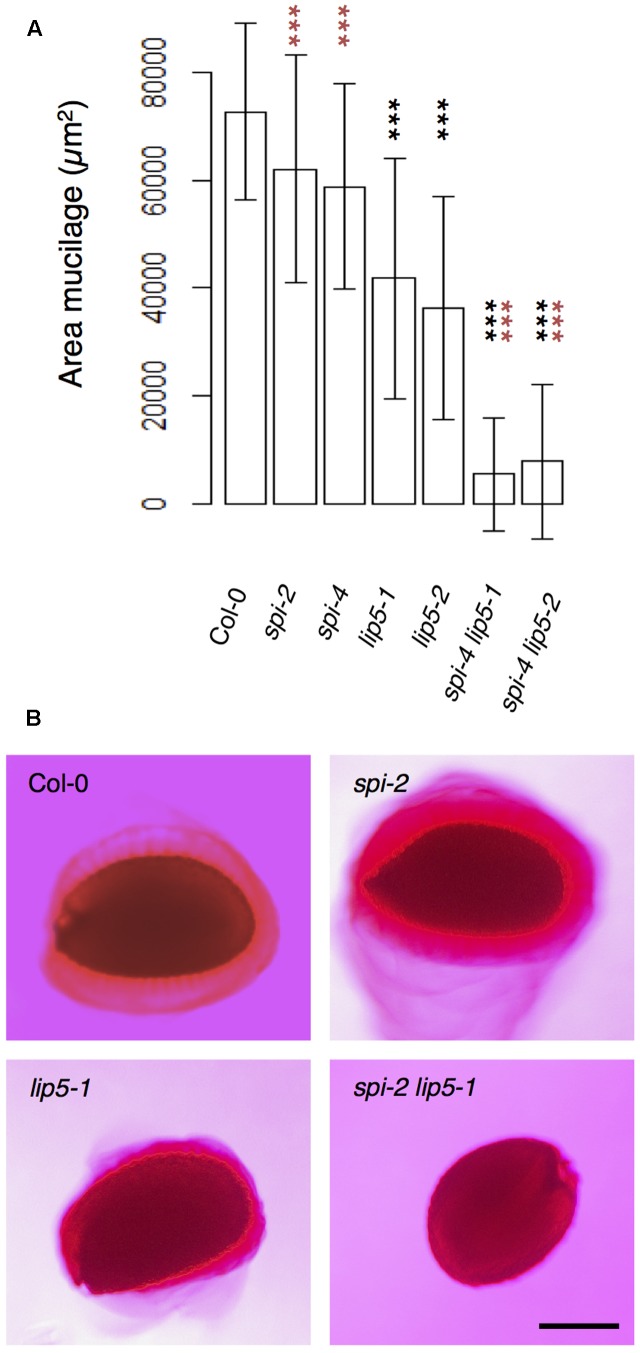
Mucilage formation in Ruthenium Red-stained seeds of *spi* and *lip5* mutants. **(A)** Mucilage areas and seed sizes are presented. Data denote the average of 30 seeds. Error bars represent SDs. Black asterisks represent statistically significant changes in comparison to Col-0 and red asterisks statistically significant changes in comparison to both *lip5* mutants (two-tailed Student’s *t*-test; ^∗∗∗^*p* < 0.001). **(B)** Representative images of Ruthenium Red-stained seeds. Scale bar: 200 μm.

To test the functional relevance of the SPI/LIP5/SKD1 interactions, we performed a genetic analysis. We focused on the seed coat mucilage phenotype because plants expressing dominant negative SKD1 produce seeds lacking seed mucilage completely (Shahriari et al., 2010a). Seed coat mucilage is enriched in polymeric sugars that are secreted from the outer seed coat cells. Its presence can be monitored by Ruthenium Red staining of whole seeds (Western et al., 2000). We found no difference between *spi* and wild-type seeds (**Figures 4A,B**). The mucilage layer of *lip5* mutant seeds was still formed, but its area was significantly smaller than in wild-type seeds. This observation is consistent with the finding that LIP5 acts as an enhancer of SKD1 ATPase activity (Haas et al., 2007). The *spi lip5* double mutant seeds exhibited a strong reduction of the mucilage layer that was significantly different to wild type as well as *lip5* single mutant seeds (**Figures 4A,B**). Apart from this mucilage phenotype we did not note any differences to *spi* mutants in *spi lip5* double mutant plants with respect to growth and morphological features. These data indicate that the secretion of seed coat mucilage requires the SKD1/SPI/LIP5 pathway and that SPI and LIP5 act synergistically.

### Synergistic Function of SPI and LIP5 in Vacuolar Transport

**FIGURE 5:**
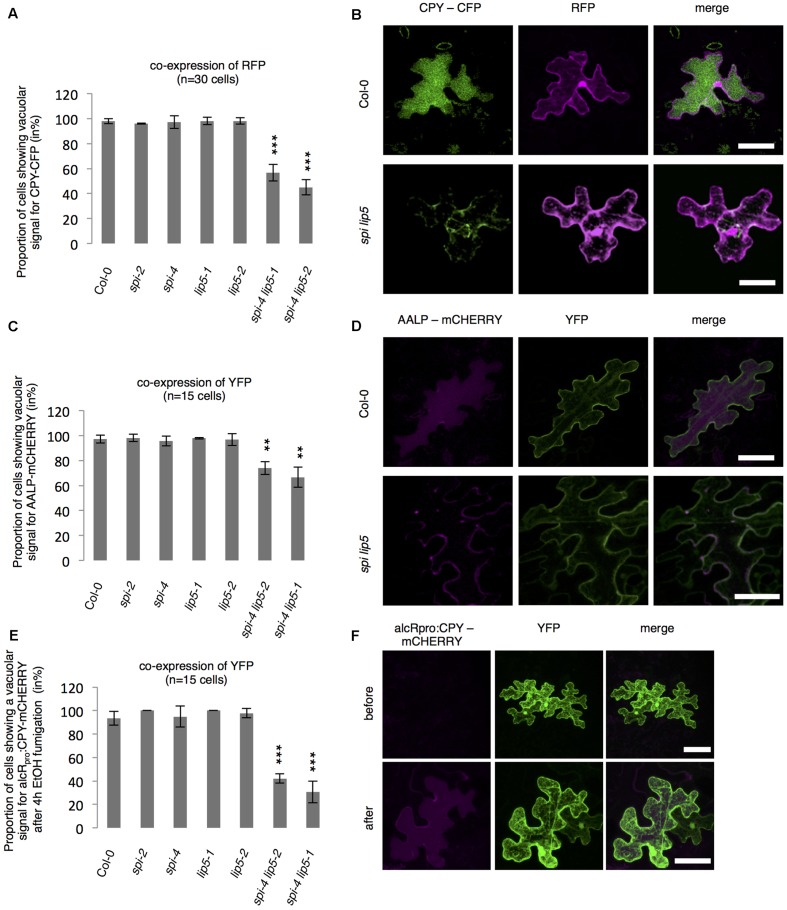
Analysis of vacuolar transport in *spi* and *lip5* mutants. **(A)** Number of cells (in %) showing a vacuolar signal for CPY-CFP in cells co-expressing free RFP. Data denote the average of three biological replicates (*n* = 30 cells each). **(B)** Representative maximum projections of CPY-CFP (column I) in transfected leaf epidermis cells of Col-0 (Upper) and *spi lip5* double mutants (Lower) co-expressing free RFP (column II) as transformation control. Column III presents the overlay picture of columns I and II. **(C)** Number of cells (in %) showing a vacuolar signal for AALP-mCHERRY in cells co-expressing free YFP. Data denote the average of three biological replicates (*n* = 15 cells each). **(D)** Representative maximum projections of AALP-mCHERRY (column I) in transfected leaf epidermis cells of Col-0 (Upper) and *spi lip5* double mutants (Lower) co-expressing free YFP (column II) as transformation control. Column III presents the overlay picture of columns I and II. **(E)** Number of cells (in %) showing vacuolar signal for alcR_pro_:CPY-mCHERRY in cells co-expressing free YFP 4 h after fumigation with 2% EtOH (related to Supplementary Figure S4). Data denote the average of three biological replicates (*n* = 15 cells each). Error bars in **A,C,E** represent SDs. Asterisks represent statistically significant changes in comparison to Col-0 (two-tailed Student’s *t*-test; ^∗∗^*p* < 0.01, ^∗∗∗^*p* < 0.001). **(F)** Representative maximum projections of alcR_pro_:CPY-mCHERRY (column I) before (Upper) and after induction of gene expression (Lower) in transfected leaf epidermis cells of Col-0 co-expressing free YFP (column II) as transformation control. Column III presents the overlay picture of columns I and II. Scale bars: 30 μm.

To test whether SPI is functionally involved in the endosomal-vacuolar transport route, we examined the intracellular localization of two soluble vacuolar enzymes CPY and AALP in *spi* mutants (Rojo et al., 2003; Shahriari et al., 2010b). Both, CPY-CFP and AALP-mCHERRY were found in the vacuole in more than 90% of the cells. Their distribution was unaffected in both *spi* and *lip5* single mutants. However, in *spi lip5* double mutants we found a severe reduction of cells displaying vacuolar CPY and AALP (**Figures 5A–D**). The vacuolar sorting defect was even more obvious after controlled induction of CPY-mCHERRY expression using an EtOH-inducible vector system (Roslan et al., 2001) (**Figures 5E,F** and Supplementary Figure S4).

**FIGURE 6:**
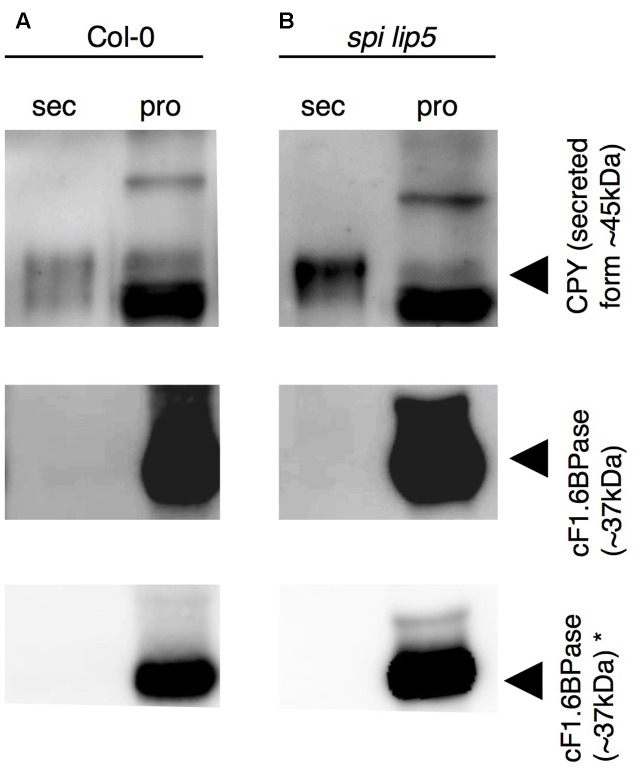
CPY is aberrantly secreted in protoplasts from *spi lip5* double mutants. Detection of CPY in total protein samples from **(A)** Col-0 and **(B)** *spi lip5*-derived leaf mesophyll protoplasts before their incubation in secretion medium (raw), the secretion medium (sec) after 60 min of incubation and the corresponding intact protoplasts fraction (pro), on α-CPY (Rojo et al., 2003) stained Western blots. The positions of CPY precursor and intermediate forms are indicated by arrowheads. cF1.6BPase was detected by α-cF1.6BPase antibody (Agrisera antibodies) staining and used as control. Please note that the contrasts of α-CPY and α-cF1.6BPase stained Western blots have been adjusted to easily visualize any protein present in the secretory fraction. Non-manipulated α-cF1.6BPase stained Western blots are presented in the lowest row (indicated by ^∗^). The same pattern of CPY and cF1.6BPase was observed in a second independent experiment.

We reasoned that the vacuolar sorting defect might result in an increased secretion similar as observed in plants expressing dominant negative SKD1 (Shahriari et al., 2010b). We therefore performed a secretion assay using Col-0 and *spi lip5* leaf mesophyll protoplasts. We compared the amounts of CPY present in the fraction of protoplasts with those secreted to the surrounding medium after 1 h of incubation (**Figure 6**). As compared to wild type, we found a stronger signal of CPY in the medium of *spi lip5* protoplasts in two independent experiments (one shown in **Figure 6**). We excluded that the CPY signals from the medium samples were caused by impurities from damaged protoplast by using α-cytoplasmic Fructose-1.6-Bisphosphatase (cF1.6BPase) as a control (**Figure 6**).

### The Absence of SPI and LIP5 Can Be Compensated by Overexpression of SKD1

**FIGURE 7:**
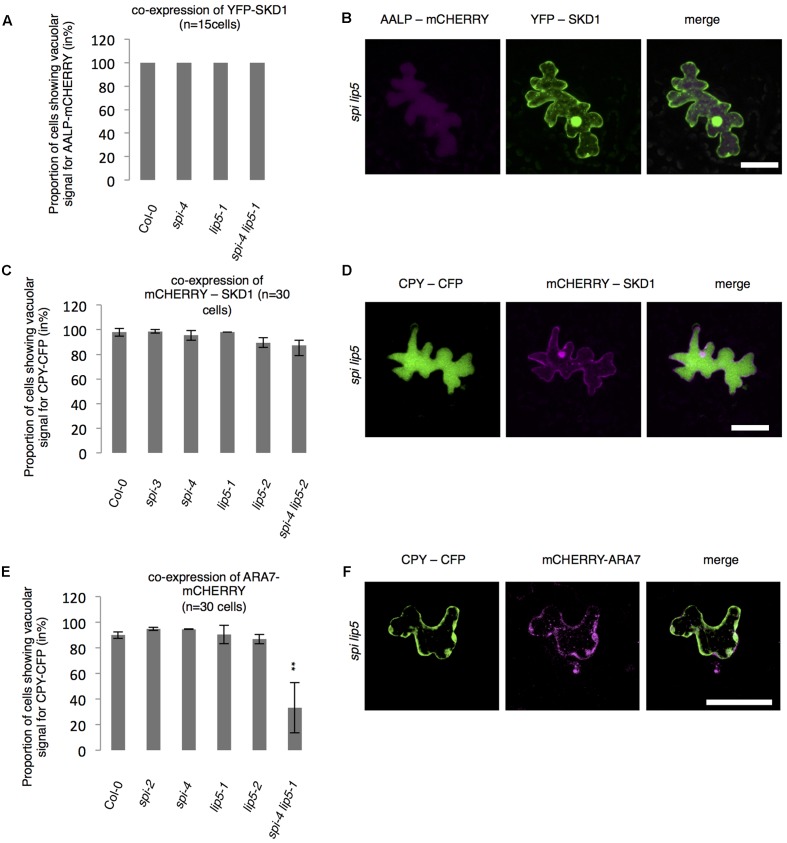
Vacuolar transport in *spi lip5* mutants can be restored by SKD1. **(A)** Number of cells (in %) showing a vacuolar signal for AALP-mCHERRY in cells co-expressing SKD1. **(B)** Representative images of AALP-mCHERRY (column I) in transfected *spi lip5* mutant leaf epidermis cells co-expressing SDK1-mCHERRY (column II). Column III presents the overlay-picture of columns I and II. Scale bar: 30 μm. **(C)** Number of cells (in %) showing a vacuolar signal for CPY-CFP in cells co-expressing SKD1. **(D)** Representative images of CPY-CFP (column I) in transfected *spi lip5* mutant leaf epidermis cells co-expressing SDK1-mCHERRY (column II). Column III presents the overlay-picture of columns I and II. Scale bar: 30 μm. **(E)** Number of cells (in %) showing a vacuolar signal for CPY-CFP in cells co-expressing mCHERRY-ARA7. Data of **A,C,E** denote the average numbers (in %) of three biological replicates. Error bars represent SDs. Asterisks represent statistically significant changes in comparison to Col-0 (two-tailed Student’s *t*-test; ^∗∗^*p* < 0.01). **(F)** Representative image of CPY-CFP (column I) in transfected *spi lip5* mutant leaf epidermis cells co-expressing mCHERRY-ARA7 (column II). Column III shows the overlay picture of columns I and II. Scale bar: 30 μm.

The finding that the *spi lip5* double mutants share many phenotypes with the dominant negative SKD1 mutants (mucilage phenotype, vacuolar transport defects, aberrant protein secretion) suggests that they may act together in promoting SKD1 activity. In this case, one would expect that the overexpression of SKD1 can compensate the reduced activation of SKD1 in *spi lip5* double mutants. We therefore tested whether overexpression of SKD1 can rescue the *spi lip5* vacuolar transport phenotype. Toward this end, we assessed the localization of AALP-mCHERRY and CPY-CFP in *spi lip5* double mutants co-expressing SKD1. The vacuolar transport of AALP was restored in 100% (**Figures 7A,B**) and of CPY-CFP in 80% of cells (**Figures 7C,D**). Co-expression of the late endosomal marker ARA7 did not restore the vacuolar transport of CPY-CFP (**Figures 7E,F**), highlighting the SKD1-specificity of the rescue. These data suggest that LIP5 and SPI regulate endosomal-vacuolar transport by acting as positive regulators of SKD1.

## Discussion

Initially *spi* has been described as a member of the class of Arabidopsis distorted mutants. In contrast to all other mutants in this class, *spi* displays no actin phenotype suggesting that it may regulate cell morphogenesis in a cytoskeleton-independent manner. As SPI encodes a BDCP homolog it was initially postulated that SPI may function in endomembrane dynamics. The finding, however, that SPI has a molecular function in the post-transcriptional regulation of mRNA stability raised the question, whether SPI has an additional role in membrane dynamics. Here, we provide genetic and molecular data that functionally link SPI and SKD1 and LIP5. A connection between the distorted phenotype and endosomal transport has not been revealed so far. Neither the *lip5* mutant nor expression of dominant negative SKD1 has been reported to trigger a distorted phenotype. Further studies might aim to investigate if there is any direct connection between these fields of research.

### BDCPs – How Do They Control Membrane Dynamics?

The common phenotypes of BDCP mutants points to their function in the control of membrane fusion and fission events at different steps along the endomembrane system (Cullinane et al., 2013). Recently, a working model was developed that proposes BDCPs to act as scaffold proteins that regulate membrane dynamics in a binding partner-dependent manner (Cullinane et al., 2013). However, only few binding partners of BDCPs have been identified (Adam-Klages et al., 1996; Segui et al., 2001; Tchernev et al., 2002; Filimonenko et al., 2010; Hocking et al., 2010) and their contribution to the pathophysiology observed in BDCP mutants was not characterized in detail so far. A possible link between BDCPs and the LIP5/SKD1 complex had been suggested by the findings that LIP5 interacts with LYST in yeast two-hybrid assays (Tchernev et al., 2002) and that over-expression of a dominant negative SKD1 protein leads to an association of LYST to membranes (Fujita et al., 2004). Our finding that SPI directly interacts not only with LIP5 but also with SKD1 strongly suggest that a functional link between BDCPs and LIP5/SKD1 is evolutionarily conserved.

### SPI Localization to MVBs by Binding to SKD1 and/or LIP5

As shown for human LYST, the SPI protein is evenly distributed in the cytoplasm (Perou et al., 1997; Fujita et al., 2004). The previous finding that LYST relocates to membranes only upon co-expression of dominant negative SKD1 (E235Q) suggests that it is either recruited under these conditions, or that it is present at endosomal membranes at low levels and becomes detectable because of the formation of large endosomal class E compartments. Consistent with both scenarios, we observe SPI interactions with LIP5 and SKD1 at endosomes in BiFC experiments. As BiFC tends to stabilize protein–protein interactions (Kerppola, 2008) a recruitment of SPI to endosomes by its binding partners is conceivable.

Membrane association of other BDCPs has been shown to depend on their binding to phospholipids. The human BDCP factor associated with neutral sphingomyelinase activation (FAN) binds directly to plasma membrane associated phosphoinositides (Haubert et al., 2007). Other BDCPs such as the mammalian homologs NBEA and lipopolysaccharide-responsive and beige-like anchor protein (LRBA) do not bind to phospholipids. Crystal structure analysis of LRBA revealed an uncommon PH domain, lacking a cluster of basic residues essential to form the classical phospholipid binding fold (Gebauer et al., 2004; Haubert et al., 2007; Lemmon, 2007). SPI protein does not appear to bind to phosphoinositides suggesting that the recruitment of SPI to membranes may be mediated by binding to other proteins.

### SPI and LIP5 Are Positive Regulators of the Protein Transport between MVBs and Plant Lytic Vacuoles

The ATPase activity of SKD1 is essential for the disassembly of the ESCRT machinery from membranes, and thereby the formation of ILVs in late endosomal compartments (Babst et al., 1998; Azmi et al., 2006; Cai et al., 2014). The depletion of SKD1 and expression of its dominant negative SKD1 versions cause the formation of aberrantly enlarged pre-vacuolar structures (class E compartments) in various species (Fujita et al., 2003; Shahriari et al., 2010b). In plants, the expression of dominant negative SKD1 causes vacuole fragmentation suggesting that the membrane transport between MVBs and the vacuole is impaired (Shahriari et al., 2010b). Although *spi* mutants exhibit fragmented vacuoles (Saedler et al., 2009), we found no obvious defects in endosomal trafficking. Neither the internalization of the styryl dye FM4-64 (Saedler et al., 2009) nor the vacuolar transport of soluble cargo proteins (this study) were affected in *spi* mutants. Thus, the cellular *spi* mutant phenotype differs to other class E (Fujita et al., 2003; Haas et al., 2007) or BDCP mutants (Faigle et al., 1998; Cornillon et al., 2002). Similar results were reported for *lip5* mutant plants (Cai et al., 2014). Although LIP5 promotes the enzymatic activity of SKD1, *lip5* mutants exhibit no global mutant phenotypes (Haas et al., 2007). However, a role of *LIP5* in MVB biogenesis was reported in the context of stress responses (Wang et al., 2014, 2015). In addition, trafficking defects of PIN proteins in roots were correlated with impaired gravitropism (Buono et al., 2016). Together these data suggest that LIP5 is a regulator of SKD1 but not an absolute requirement for SKD1 activity. Our genetic studies, showing a redundant function of LIP5 and SPI suggest that the two proteins act together in the regulation of SKD1. As the additional expression of SKD1 can overcome the requirement of LIP5 and SPI it is likely that SKD1 acts genetically downstream of LIP5 and SPI. It is conceivable that SPI acts together with LIP5 as a positive regulator of SKD1. This conclusion appears to be in conflict with data obtained in human cell culture systems. Here, overexpression of SKD1 was not sufficient to restore the giant lysosome phenotype in LYST deficient fibroblasts suggesting that LYST acts downstream of SKD1-mediated membrane transport (Fujita et al., 2004). This suggests that the molecular function we describe for the BDCP SPI is mechanistically different to that of BDCPs in humans. An alternative explanation would be that a rescue of protein/membrane trafficking by additional expression of SKD1 is independent of a rescue of endosomal sizes.

## Materials and Methods

### Plant Material, Protoplast Isolation, Growth Conditions, and Mucilage Staining

*Arabidopsis thaliana spi-2* (GK_205G08*), spi-3* (SALK_065311), *spi-4* (GK_420D09), *lip5-1* (SAIL_854F08) and *lip5*-2 (GK_351B10) have been previously described (Haas et al., 2007; Wang et al., 2014); and were obtained from the National Arabidopsis Stock Center. Positions of T-DNA insertions were confirmed by sequencing the flanking genomic regions. Seeds were grown on soil under long day conditions (22°C; 110 μmol m^-2^ s^-1^). Ruthenium Red Staining of seed coat mucilage was performed in H_2_O (+0.1% Tween20) as described (Western et al., 2000). Isolation of protoplasts from Arabidopsis leaves was performed according to the Tape-Arabidopsis Sandwich method (Wu et al., 2009). Fifty microliters protoplasts were harvested and washed with 150 μl of W5 solution (154 mM NaCl, 125 mM CaCl_2_, 5 mM KCl, 5 mM glucose, and 2 mM MES, pH 5.7) in a 1.5 ml Eppendorf tube at 100*g* for 5 min. For determination of protein secretion 50 μl of washed protoplasts were incubated in 50 μl of W5 solution for 60 min at room temperature in the dark. After centrifugation (5 min, 100*g*), supernatant (secretion fraction) and intact protoplasts were divided and boiled in protein loading buffer for further analysis.

### Plasmids

Coding sequences (CDS) of SPI-PBW (AT1G03060) was described before (Steffens et al., 2015). The CDS of SPI-PB and AALP (AT5G60360) were amplified from Col-0 cDNA. Primer sequences are provided in Supplementary Table S1. Entry clones of SKD1 (AT2G27600), LIP5 (AT4G26750), CPY (AT3G10410), ARA6 (AT3G54840) and ARA7 (AT4G19640) were published before (Shahriari et al., 2010b). All constructs were confirmed by sequencing. A list of GATEWAY^®^-vectors used in this study is provided in Supplementary Table S2.

### Protein–Protein/Protein–Lipid Interaction Assays and Protein Purifications

Yeast two-hybrid assays were performed as described (Gietz et al., 1995). Interactions were analyzed on dropout media lacking leucine (L), tryptophan (W) and histidine (H) [+20 mM 3-Amino-1,2,3-Triazole (3AT)]. For biochemical assays GST-SPI-PBW-His_6_, GST and His_6_-tagged SKD1 were expressed and purified as described before (Steffens et al., 2014). Lysis of bacteria expressing GST-SPI-PBW-His6 was performed in STE-Buffer (10 mM Tris pH8.0, 150 mM NaCl, 1 mM EDTA). After centrifugation of the lysates, PBS (pH 7.5) was used for washing and elution according to (Steffens et al., 2014). GST-SPI was eluted in PBS, supplemented with 40 mM reduced L-glutathione. His_6_-fusions were eluted in Tris-Elution buffer (100 mM Tris pH 8.0, 150 mM NaCl) supplemented with 500 mM imidazole. For co-precipitation and protein-lipid binding assays (Echelon bioscience; performed with 1.5 μg purified protein according to the manufacturers instructions), purified proteins were concentrated using Amicons Ultra-4 Centrifugal Filters (Merck Millipore) and dialyzed (Xpress Micro Dialyzer Cartridge MD300; Scienova) against Tris-Elution Buffer. Purifications and co-precipitations were analyzed by immunoblotting (Steffens et al., 2014). HA- and 3xFLAG fusions were (co)-immunoprecipitated using antibody conjugated μ-columns according to the manufacturer’s instructions (Miltenyi; μMACS^TM^) from total protein lysates derived from infiltrated *N. benthamiana* leaves. Bound proteins were eluted and analyzed by immunoblotting, using either a monoclonal horseradish peroxidase (HRP) coupled with anti-HA antibody from rat (Roche; 1:1000 dilution) or a primary monoclonal anti-FLAG antibody from mouse (Sigma–Aldrich; 1:2000 dilution) and a secondary anti-mouse antibody from goat coupled to HRP (Sigma–Aldrich; 1:10,000).

### Transient Expression in Plants, Induction Conditions and Confocal Laser Scanning Microscopy (CLSM)

*Nicotiana benthamiana* leaves were transformed as described (Yang et al., 2000). Biolistic transformation of Arabidopsis leaves was performed as described (Mathur et al., 2003) and analyzed after 12 to 16 h by Confocal Laser Scanning Microscopy (CLSM). CLSM was performed as described (Steffens et al., 2014). Expression of alcR_pro_-fusion proteins (pCAMPARI-vector) was induced by fumigation of transfected leaves with 2% ethanol (v/v) for 4 h (Roslan et al., 2001). Before induction of protein expression, transfected cells were identified by the presence of constantly co-expressed free YFP. To ensure comparisons between equally strong transfected cells, laser intensities and exposure times were fixed.

## Accession Numbers

Sequence data from this article can be found in the EMBL/GenBank data libraries under accession numbers: AT1G03060 (SPI), AT5G60360 (AALP), AT2G27600 (SKD1), AT4G26750 (LIP5), AT3G10410 (CPY), AT3G54840 (ARA6), and AT4G19640 (ARA7).

## Author Contributions

AS and MH designed this study. AS performed the research and analyzed the data. AS and MJ generated plasmids and established mutant lines. AS and MH wrote the paper. MH directed the project.

## Conflict of Interest Statement

The authors declare that the research was conducted in the absence of any commercial or financial relationships that could be construed as a potential conflict of interest.

## References

[B1] Adam-Klages S. Adam D. Wiegmann K. Struve S. Kolanus W. Schneider-Mergener J. (1996). FAN, a novel WD-repeat protein, couples the p55 TNF-receptor to neutral sphingomyelinase. *Cell* 86 937–947. 10.1016/S0092-8674(00)80169-5 8808629

[B2] Azmi I. Davies B. Dimaano C. Payne J. Eckert D. Babst M. (2006). Recycling of ESCRTs by the AAA-ATPase Vps4 is regulated by a conserved VSL region in Vta1. *J. Cell Biol.* 172 705–717. 10.1083/jcb.200508166 16505166 PMC2063703

[B3] Babst M. (2005). A protein’s final ESCRT. *Traffic* 6 2–9. 10.1111/j.1600-0854.2004.00246.x 15569240

[B4] Babst M. Wendland B. Estepa E. J. Emr S. D. (1998). The Vps4p AAA ATPase regulates membrane association of a Vps protein complex required for normal endosome function. *EMBO J.* 17 2982–2993. 10.1093/emboj/17.11.2982 9606181 PMC1170638

[B5] Barbosa M. D. Nguyen Q. A. Tchernev V. T. Ashley J. A. Detter J. C. Blaydes S. M. (1996). Identification of the homologous beige and Chediak-Higashi syndrome genes. *Nature* 382 262–265. 10.1038/382262a0 8717042 PMC2893578

[B6] Brandt E. J. Elliott R. W. Swank R. T. (1975). Defective lysosomal enzyme secretion in kidneys of Chediak-Higashi (beige) mice. *J. Cell Biol.* 67 774–788. 10.1083/jcb.67.3.774 408 PMC2111673

[B7] Buono R. A. Paez-Valencia J. Miller N. D. Goodman K. Spitzer C. Spalding E. P. (2016). Role of SKD1 regulators LIP5 and IST1-LIKE1 in endosomal sorting and plant development. *Plant Physiol.* 171 251–264. 10.1104/pp.16.00240 26983994 PMC4854716

[B8] Cai Y. Zhuang X. Gao C. Wang X. Jiang L. (2014). The Arabidopsis endosomal sorting complex required for transport III regulates internal vesicle formation of the prevacuolar compartment and is required for plant development. *Plant Physiol.* 165 1328–1343. 10.1104/pp.114.238378 24812106 PMC4081340

[B9] Cornillon S. Dubois A. Bruckert F. Lefkir Y. Marchetti A. Benghezal M. (2002). Two members of the beige/CHS (BEACH) family are involved at different stages in the organization of the endocytic pathway in *Dictyostelium*. *J. Cell Sci.* 115 737–744. 11865029 10.1242/jcs.115.4.737

[B10] Cullinane A. R. Schaffer A. A. Huizing M. (2013). The BEACH is hot: a LYST of emerging roles for BEACH-domain containing proteins in human disease. *Traffic* 14 749–766. 10.1111/tra.12069 23521701 PMC3761935

[B11] de Souza N. Vallier L. G. Fares H. Greenwald I. (2007). SEL-2, the *C. elegans* neurobeachin/LRBA homolog, is a negative regulator of lin-12/Notch activity and affects endosomal traffic in polarized epithelial cells. *Development* 134 691–702. 10.1242/dev.02767 17215302

[B12] del Pino I. Paarmann I. Karas M. Kilimann M. W. Betz H. (2011). The trafficking proteins Vacuolar Protein Sorting 35 and Neurobeachin interact with the glycine receptor beta-subunit. *Biochem. Biophys. Res. Commun.* 412 435–440. 10.1016/j.bbrc.2011.07.110 21821005

[B13] Ebine K. Fujimoto M. Okatani Y. Nishiyama T. Goh T. Ito E. (2011). A membrane trafficking pathway regulated by the plant-specific RAB GTPase ARA6. *Nat. Cell Biol.* 13 853–859. 10.1038/ncb2270 21666683

[B14] Faigle W. Raposo G. Tenza D. Pinet V. Vogt A. B. Kropshofer H. (1998). Deficient peptide loading and MHC class II endosomal sorting in a human genetic immunodeficiency disease: the Chediak-Higashi syndrome. *J. Cell Biol.* 141 1121–1134. 10.1083/jcb.141.5.1121 9606205 PMC2137185

[B15] Ferguson K. M. Lemmon M. A. Schlessinger J. Sigler P. B. (1995). Structure of the high affinity complex of inositol trisphosphate with a phospholipase C pleckstrin homology domain. *Cell* 83 1037–1046. 10.1016/0092-8674(95)90219-8 8521504

[B16] Filimonenko M. Isakson P. Finley K. D. Anderson M. Jeong H. Melia T. J. (2010). The selective macroautophagic degradation of aggregated proteins requires the PI3P-binding protein Alfy. *Mol. Cell.* 38 265–279. 10.1016/j.molcel.2010.04.007 20417604 PMC2867245

[B17] Fujita H. Umezuki Y. Imamura K. Ishikawa D. Uchimura S. Nara A. (2004). Mammalian class E Vps proteins, SBP1 and mVps2/CHMP2A, interact with and regulate the function of an AAA-ATPase SKD1/Vps4B. *J. Cell Sci.* 117 2997–3009. 10.1242/jcs.01170 15173323

[B18] Fujita H. Yamanaka M. Imamura K. Tanaka Y. Nara A. Yoshimori T. (2003). A dominant negative form of the AAA ATPase SKD1/VPS4 impairs membrane trafficking out of endosomal/lysosomal compartments: class E vps phenotype in mammalian cells. *J. Cell Sci.* 116 401–414. 10.1242/jcs.00213 12482925

[B19] Gao C. Zhuang X. Cui Y. Fu X. He Y. Zhao Q. (2015). Dual roles of an *Arabidopsis* ESCRT component FREE1 in regulating vacuolar protein transport and autophagic degradation. *Proc. Natl. Acad. Sci. U.S.A.* 112 1886–1891. 10.1073/pnas.1421271112 25624505 PMC4330779

[B20] Gebauer D. Li J. Jogl G. Shen Y. Myszka D. G. Tong L. (2004). Crystal structure of the PH-BEACH domains of human LRBA/BGL. *Biochemistry* 43 14873–14880. 10.1021/bi049498y 15554694

[B21] Gerald N. J. Siano M. De Lozanne A. (2002). The Dictyostelium LvsA protein is localized on the contractile vacuole and is required for osmoregulation. *Traffic* 3 50–60. 10.1034/j.1600-0854.2002.30107.x 11872142

[B22] Gietz R. D. Schiestl R. H. Willems A. R. Woods R. A. (1995). Studies on the transformation of intact yeast cells by the LiAc/SS-DNA/PEG procedure. *Yeast* 11 355–360. 10.1002/yea.320110408 7785336

[B23] Haas T. J. Sliwinski M. K. Martinez D. E. Preuss M. Ebine K. Ueda T. (2007). The *Arabidopsis* AAA ATPase SKD1 is involved in multivesicular endosome function and interacts with its positive regulator LYST-INTERACTING PROTEIN5. *Plant Cell* 19 1295–1312. 10.1105/tpc.106.049346 17468262 PMC1913750

[B24] Harris E. Wang N. Wu W. L. Weatherford A. De Lozanne A. Cardelli J. (2002). Dictyostelium LvsB mutants model the lysosomal defects associated with Chediak-Higashi syndrome. *Mol. Biol. Cell* 13 656–669. 10.1091/mbc.01-09-0454 11854420 PMC65657

[B25] Haubert D. Gharib N. Rivero F. Wiegmann K. Hosel M. Kronke M. (2007). PtdIns(4,5)P-restricted plasma membrane localization of FAN is involved in TNF-induced actin reorganization. *EMBO J.* 26 3308–3321. 10.1038/sj.emboj.7601778 17599063 PMC1933409

[B26] Hocking L. J. Mellis D. J. McCabe P. S. Helfrich M. H. Rogers M. J. (2010). Functional interaction between sequestosome-1/p62 and autophagy-linked FYVE-containing protein WDFY3 in human osteoclasts. *Biochem. Biophys. Res. Commun.* 402 543–548. 10.1016/j.bbrc.2010.10.076 20971078

[B27] Hulskamp M. Misra S. Jurgens G. (1994). Genetic dissection of trichome cell development in Arabidopsis. *Cell* 76 555–566. 10.1016/0092-8674(94)90118-X 8313475

[B28] Hurley J. H. Emr S. D. (2006). The ESCRT complexes: structure and mechanism of a membrane-trafficking network. *Annu. Rev. Biophys. Biomol. Struct.* 35 277–298. 10.1146/annurev.biophys.35.040405.102126 16689637 PMC1648078

[B29] Introne W. Boissy R. E. Gahl W. A. (1999). Clinical, molecular, and cell biological aspects of Chediak-Higashi syndrome. *Mol. Genet. Metab.* 68 283–303. 10.1006/mgme.1999.2927 10527680

[B30] Introne W. J. Westbroek W. Golas G. A. Adams D. (1993). “Chediak-Higashi Syndrome,” in *SourceGeneReviews^®^* [Internet], eds Adam M. P. Ardinger H. H. Pagon R. A. Wallace S. E. Bean L. J. H. Mefford H. C. (Seattle, WA: University of Washington), 1993–2017. 20301751

[B31] Kaplan J. De Domenico I. Ward D. M. (2008). Chediak-Higashi syndrome. *Curr. Opin. Hematol.* 15 22–29. 10.1097/MOH.0b013e3282f2bcce 18043242

[B32] Katsiarimpa A. Munoz A. Kalinowska K. Uemura T. Rojo E. Isono E. (2014). The ESCRT-III-interacting deubiquitinating enzyme AMSH3 is essential for degradation of ubiquitinated membrane proteins in *Arabidopsis thaliana*. *Plant Cell Physiol.* 55 727–736. 10.1093/pcp/pcu019 24486765

[B33] Kerppola T. K. (2008). Bimolecular fluorescence complementation (BiFC) analysis as a probe of protein interactions in living cells. *Annu. Rev. Biophys.* 37 465–487. 10.1146/annurev.biophys.37.032807.125842 18573091 PMC2829326

[B34] Khodosh R. Augsburger A. Schwarz T. L. Garrity P. A. (2006). Bchs, a BEACH domain protein, antagonizes Rab11 in synapse morphogenesis and other developmental events. *Development* 133 4655–4665. 10.1242/dev.02650 17079274

[B35] Landsberg M. J. Vajjhala P. R. Rothnagel R. Munn A. L. Hankamer B. (2009). Three-dimensional structure of AAA ATPase Vps4: advancing structural insights into the mechanisms of endosomal sorting and enveloped virus budding. *Structure* 17 427–437. 10.1016/j.str.2008.12.020 19278657

[B36] Lata S. Schoehn G. Jain A. Pires R. Piehler J. Gottlinger H. G. (2008). Helical structures of ESCRT-III are disassembled by VPS4. *Science* 321 1354–1357. 10.1126/science.1161070 18687924 PMC2758909

[B37] Lemmon M. A. (2007). Pleckstrin homology (PH) domains and phosphoinositides. *Biochem. Soc. Symp.* 74 81–93. 10.1042/BSS2007c08 PMC3777418 17233582

[B38] Mathur J. Mathur N. Kirik V. Kernebeck B. Srinivas B. P. Hulskamp M. (2003). *Arabidopsis CROOKED* encodes for the smallest subunit of the ARP2/3 complex and controls cell shape by region specific fine F-actin formation. *Development* 130 3137–3146. 10.1242/dev.00549 12783786

[B39] Matile P. (1968). Lysosomes of root tip cells in corn seedlings. *Planta* 79 181–196. 10.1007/BF00396026 24522868

[B40] Mesquita J. F. (1969). Electron microscope study of the origin and development of the vacuoles in root-tip cells of *Lupinus albus* L. *J. Ultrastruct. Res.* 26 242–250. 10.1016/S0022-5320(69)80004-3 5776619

[B41] Nagle D. L. Karim M. A. Woolf E. A. Holmgren L. Bork P. Misumi D. J. (1996). Identification and mutation analysis of the complete gene for Chediak-Higashi syndrome. *Nat. Genet.* 14 307–311. 10.1038/ng1196-307 8896560

[B42] Neer E. J. Schmidt C. J. Nambudripad R. Smith T. F. (1994). The ancient regulatory-protein family of WD-repeat proteins. *Nature* 371 297–300. 10.1038/371297a0 8090199

[B43] Perou C. M. Leslie J. D. Green W. Li L. Ward D. M. Kaplan J. (1997). The Beige/Chediak-Higashi syndrome gene encodes a widely expressed cytosolic protein. *J. Biol. Chem.* 272 29790–29794. 10.1074/jbc.272.47.29790 9368050

[B44] Perou C. M. Moore K. J. Nagle D. L. Misumi D. J. Woolf E. A. McGrail S. H. (1996). Identification of the murine beige gene by YAC complementation and positional cloning. *Nat. Genet.* 13 303–308. 10.1038/ng0796-303 8673129

[B45] Pesch M. Schultheiss I. Digiuni S. Uhrig J. F. Hulskamp M. (2013). Mutual control of intracellular localisation of the patterning proteins AtMYC1, GL1 and TRY/CPC in *Arabidopsis*. *Development* 140 3456–3467. 10.1242/dev.094698 23900543

[B46] Raymond C. K. Howald-Stevenson I. Vater C. A. Stevens T. H. (1992). Morphological classification of the yeast vacuolar protein sorting mutants: evidence for a prevacuolar compartment in class E vps mutants. *Mol. Biol. Cell* 3 1389–1402. 10.1091/mbc.3.12.1389 1493335 PMC275707

[B47] Richardson L. G. Howard A. S. Khuu N. Gidda S. K. McCartney A. Morphy B. J. (2011). Protein-protein interaction network and subcellular localization of the *Arabidopsis thaliana* ESCRT Machinery. *Front. Plant Sci.* 2:20. 10.3389/fpls.2011.00020 22639582 PMC3355721

[B48] Rojo E. Zouhar J. Carter C. Kovaleva V. Raikhel N. V. (2003). A unique mechanism for protein processing and degradation in *Arabidopsis thaliana*. *Proc. Natl. Acad. Sci. U.S.A.* 100 7389–7394. 10.1073/pnas.1230987100 12773619 PMC165885

[B49] Roslan H. A. Salter M. G. Wood C. D. White M. R. Croft K. P. Robson F. (2001). Characterization of the ethanol-inducible alc gene-expression system in *Arabidopsis thaliana*. *Plant J.* 28 225–235. 10.1046/j.1365-313X.2001.01146.x 11722766

[B50] Sachse M. Strous G. J. Klumperman J. (2004). ATPase-deficient hVPS4 impairs formation of internal endosomal vesicles and stabilizes bilayered clathrin coats on endosomal vacuoles. *J. Cell Sci.* 117 1699–1708. 10.1242/jcs.00998 15075231

[B51] Saedler R. Jakoby M. Marin B. Galiana-Jaime E. Hulskamp M. (2009). The cell morphogenesis gene *SPIRRIG* in Arabidopsis encodes a WD/BEACH domain protein. *Plant J.* 59 612–621. 10.1111/j.1365-313X.2009.03900.x 19392685

[B52] Saint-Jore-Dupas C. Gomord V. Paris N. (2004). Protein localization in the plant Golgi apparatus and the trans-Golgi network. *Cell Mol. Life Sci.* 61 159–171. 10.1007/s00018-003-3354-7 14745495 PMC11138554

[B53] Scheuring D. Kunzl F. Viotti C. Yan M. S. Jiang L. Schellmann S. (2012). Ubiquitin initiates sorting of Golgi and plasma membrane proteins into the vacuolar degradation pathway. *BMC Plant Biol.* 12:164. 10.1186/1471-2229-12-164 22970698 PMC3534617

[B54] Scheuring D. Viotti C. Kruger F. Kunzl F. Sturm S. Bubeck J. (2011). Multivesicular bodies mature from the *trans*-Golgi network/early endosome in *Arabidopsis*. *Plant Cell* 23 3463–3481. 10.1105/tpc.111.086918 21934143 PMC3203422

[B55] Schwab B. Mathur J. Saedler R. Schwarz H. Frey B. Scheidegger C. (2003). Regulation of cell expansion by the DISTORTED genes in *Arabidopsis thaliana*: actin controls the spatial organization of microtubules. *Mol. Genet. Genomics* 269 350–360. 10.1007/s00438-003-0843-1 12690443

[B56] Segui B. Cuvillier O. Adam-Klages S. Garcia V. Malagarie-Cazenave S. Leveque S. (2001). Involvement of FAN in TNF-induced apoptosis. *J. Clin. Invest.* 108 143–151. 10.1172/JCI11498 11435466 PMC209337

[B57] Shahriari M. Hulskamp M. Schellmann S. (2010a). Seeds of Arabidopsis plants expressing dominant-negative AtSKD1 under control of the GL2 promoter show a transparent testa phenotype and a mucilage defect. *Plant Signal. Behav.* 5 1308–1310. 10.4161/psb.5.10.13134 20930567 PMC3115375

[B58] Shahriari M. Keshavaiah C. Scheuring D. Sabovljevic A. Pimpl P. Hausler R. E. (2010b). The AAA-type ATPase AtSKD1 contributes to vacuolar maintenance of *Arabidopsis thaliana*. *Plant J.* 64 71–85. 10.1111/j.1365-313X.2010.04310.x 20663085

[B59] Shim S. Merrill S. A. Hanson P. I. (2008). Novel interactions of ESCRT-III with LIP5 and VPS4 and their implications for ESCRT-III disassembly. *Mol. Biol. Cell* 19 2661–2672. 10.1091/mbc.E07-12-1263 18385515 PMC2397308

[B60] Spitzer C. Reyes F. C. Buono R. Sliwinski M. K. Haas T. J. Otegui M. S. (2009). The ESCRT-related CHMP1A and B proteins mediate multivesicular body sorting of auxin carriers in *Arabidopsis* and are required for plant development. *Plant Cell* 21 749–766. 10.1105/tpc.108.064865 19304934 PMC2671707

[B61] Spritz R. A. (1998). Genetic defects in Chediak-Higashi syndrome and the beige mouse. *J. Clin. Immunol.* 18 97–105. 10.1023/A:1023247215374 9533653

[B62] Steffens A. Brautigam A. Jakoby M. Hulskamp M. (2015). The BEACH domain protein SPIRRIG is essential for Arabidopsis salt stress tolerance and functions as a regulator of transcript stabilization and localization. *PLOS Biol.* 13:e1002188. 10.1371/journal.pbio.1002188 26133670 PMC4489804

[B63] Steffens A. Jaegle B. Tresch A. Hulskamp M. Jakoby M. (2014). Processing-body movement in Arabidopsis depends on an interaction between myosins and DECAPPING PROTEIN1. *Plant Physiol.* 164 1879–1892. 10.1104/pp.113.233031 24525673 PMC3982750

[B64] Szymanski D. B. Marks M. D. Wick S. M. (1999). Organized F-actin is essential for normal trichome morphogenesis in Arabidopsis. *Plant Cell* 11 2331–2347. 10.1105/tpc.11.12.2331 10590162 PMC144140

[B65] Tchernev V. T. Mansfield T. A. Giot L. Kumar A. M. Nandabalan K. Li Y. (2002). The Chediak-Higashi protein interacts with SNARE complex and signal transduction proteins. *Mol. Med.* 8 56–64. 11984006 PMC2039936

[B66] Teh O. K. Hatsugai N. Tamura K. Fuji K. Tabata R. Yamaguchi K. (2015). BEACH-domain proteins act together in a cascade to mediate vacuolar protein trafficking and disease resistance in *Arabidopsis*. *Mol. Plant* 8 389–398. 10.1016/j.molp.2014.11.015 25618824

[B67] Ueda T. Uemura T. Sato M. H. Nakano A. (2004). Functional differentiation of endosomes in Arabidopsis cells. *Plant J.* 40 783–789. 10.1111/j.1365-313X.2004.02249.x 15546360

[B68] van Balkom B. W. Boone M. Hendriks G. Kamsteeg E. J. Robben J. H. Stronks H. C. (2009). LIP5 interacts with aquaporin 2 and facilitates its lysosomal degradation. *J. Am. Soc. Nephrol.* 20 990–1001. 10.1681/ASN.2008060648 19357255 PMC2678037

[B69] Vermeer J. E. van Leeuwen W. Tobena-Santamaria R. Laxalt A. M. Jones D. R. Divecha N. (2006). Visualization of PtdIns3P dynamics in living plant cells. *Plant J.* 47 687–700. 10.1111/j.1365-313X.2006.02830.x 16856980

[B70] Viotti C. Kruger F. Krebs M. Neubert C. Fink F. Lupanga U. (2013). The endoplasmic reticulum is the main membrane source for biogenesis of the lytic vacuole in *Arabidopsis*. *Plant Cell* 25 3434–3449. 10.1105/tpc.113.114827 24014545 PMC3809542

[B71] Voigt B. Timmers A. C. Samaj J. Hlavacka A. Ueda T. Preuss M. (2005). Actin-based motility of endosomes is linked to the polar tip growth of root hairs. *Eur. J. Cell Biol.* 84 609–621. 10.1016/j.ejcb.2004.12.029 16032929

[B72] Wang F. Shang Y. Fan B. Yu J. Q. Chen Z. (2014). Arabidopsis LIP5, a positive regulator of multivesicular body biogenesis, is a critical target of pathogen-responsive MAPK cascade in plant basal defense. *PLOS Pathog.* 10:e1004243. 10.1371/journal.ppat.1004243 25010425 PMC4092137

[B73] Wang F. Yang Y. Wang Z. Zhou J. Fan B. Chen Z. (2015). A critical role of lyst-interacting protein5, a positive regulator of multivesicular body biogenesis, in plant responses to heat and salt stresses. *Plant Physiol.* 169 497–511. 10.1104/pp.15.00518 26229051 PMC4577394

[B74] Wang X. Herberg F. W. Laue M. M. Wullner C. Hu B. Petrasch-Parwez E. (2000). Neurobeachin: A protein kinase A-anchoring, beige/Chediak-higashi protein homolog implicated in neuronal membrane traffic. *J. Neurosci.* 20 8551–8565. 11102458 10.1523/JNEUROSCI.20-23-08551.2000 PMC6773050

[B75] Ward D. M. Griffiths G. M. Stinchcombe J. C. Kaplan J. (2000). Analysis of the lysosomal storage disease Chediak-Higashi syndrome. *Traffic* 1 816–822. 10.1034/j.1600-0854.2000.011102.x 11208072

[B76] Western T. L. Skinner D. J. Haughn G. W. (2000). Differentiation of mucilage secretory cells of the Arabidopsis seed coat. *Plant Physiol.* 122 345–356. 10.1104/pp.122.2.345 10677428 PMC58872

[B77] Wu F. H. Shen S. C. Lee L. Y. Lee S. H. Chan M. T. Lin C. S. (2009). Tape-*Arabidopsis* Sandwich - a simpler *Arabidopsis* protoplast isolation method. *Plant Methods* 5:16. 10.1186/1746-4811-5-16 19930690 PMC2794253

[B78] Wu W. I. Yajnik J. Siano M. De Lozanne A. (2004). Structure-function analysis of the BEACH protein LvsA. *Traffic* 5 346–355. 10.1111/j.1600-0854.2004.00177.x 15086784

[B79] Yang Y. Li R. Qi M. (2000). In vivo analysis of plant promoters and transcription factors by agroinfiltration of tobacco leaves. *Plant J.* 22 543–551. 10.1046/j.1365-313x.2000.00760.x 10886774

[B80] Zhang C. Hicks G. R. Raikhel N. V. (2014). Plant vacuole morphology and vacuolar trafficking. *Front. Plant Sci.* 5:476. 10.3389/fpls.2014.00476 25309565 PMC4173805

[B81] Zhao H. Boissy Y. L. Abdel-Malek Z. King R. A. Nordlund J. J. Boissy R. E. (1994). On the analysis of the pathophysiology of Chediak-Higashi syndrome. Defects expressed by cultured melanocytes. *Lab. Invest.* 71 25–34. 8041115

[B82] Zhao H. Wang X. Zhu D. Cui S. Li X. Cao Y. (2012). A single amino acid substitution in IIIf subfamily of basic helix-loop-helix transcription factor AtMYC1 leads to trichome and root hair patterning defects by abolishing its interaction with partner proteins in *Arabidopsis*. *J. Biol. Chem.* 287 k14109–14121. 10.1074/jbc.M111.280735 22334670 PMC3340177

[B83] Zimmermann I. M. Heim M. A. Weisshaar B. Uhrig J. F. (2004). Comprehensive identification of *Arabidopsis thaliana* MYB transcription factors interacting with R/B-like BHLH proteins. *Plant J.* 40 22–34. 10.1111/j.1365-313X.2004.02183.x 15361138

