# Supplementary material for: Physical, Functional and Genetic Interactions between the BEACH Domain Protein SPIRRIG and LIP5 and SKD1 and Its Role in Endosomal Trafficking to the Vacuole in Arabidopsis
Source: Front Plant Sci. 2017 Nov 20;8:1969. doi: 10.3389/fpls.2017.01969 (PMC5701936; doi:10.3389/fpls.2017.01969)
Supplement: Supplementary file 1 [file Table_1.DOCX]

**Table S1**: Sequences of primers used in this study.

| **Primer** | **Sequence (5´to 3´)** |
| --- | --- |
| SPI-PB fw | GGGGACAAGTTTGTACAAAAAAGCAGGCTCAATGAAATGGGCAACATTGCTTAAGGGGACTGAA |
| SPI-PB rev | GGGGACCACTTTGTACAAGAAAGCTGGGTTTTATTCGTGAGGAACTAGAT |
| AALP fw | GGGGACAAGTTTGTACAAAAAAGCAGGCTTAATGTCTGCGAAAACAATCCT |
| AALP rev | GGGGACCACTTTGTACAAGAAAGCTGGGTTTAAAGCCACAACGGGGTATG |
| J1449 | ccccGTCGACGATATTCTCCTGCACACAGC |
| J1450 | ccccgtcgaCGTCCTCTCCAAATGAAATGAAC |

**Table S2.** List of GATEWAY vectors used in this study.

| **Vector** | **Application** |
| --- | --- |
| pENSG/  pEXSG-YFP | 35S promoter driven expression of N- or C-terminal YFP-fusions *in planta* (Feys et al., 2005) |
| pAMARENA/  pAUBERGINE | 35S promoter driven expression of N- or C-terminal mCHERRY fusions *in planta* (Marc Jakoby, GenBank ID: FR695428) |
| pJ2b 3xFLAG | 35S promoter driven expression of N-terminal FLAG-fusions *in planta* (kindly received from ImreSommsich and BekirÜlker) |
| pBaTL-HA | 35S promoter driven expression of C-terminal HA-fusions *in planta* (Joachim Uhrig, unpublished) |
| pCL112/ pCL113 | 35S promoter driven expression of N-terminal YFP_N_/YFP_C_ fusions *in planta*(Joachim Uhrig, unpublished) |
| pGEX2TM-GW | Bacterial expression of N-terminal GST and C-terminal His_6_-fusions (kindly received from Imre Sommsich and Bekir Ülker) |
| pDEST17 | Bacterial expression of N-terminal His_6_-fusions (Invitrogen) |
| pAS/pACT | Expression of proteins N-terminally fused to the GAL4 Binding Domain / Activation Domain in yeast (Clontech) |
| pCAMPARI | The 35S promoter from pAUBERGINE was replaced with the UBQ10 promoter (AscI/XhoI) to produce pAUBERGINEpUBI. The alcR/alcA containing fragment was amplified using primers J1449 /J1450, cloned into pJET2.1 (Thermo Scientific), sequenced and SgrDI digested. The alcR/alcA fragment was ligated downstream of the UBQ10 promoter into the XhoI site upstream of a GATEWAY cassette–mCHERRY to yield pCAMPARI. |
