# Supplementary material for: Physical, Functional and Genetic Interactions between the BEACH Domain Protein SPIRRIG and LIP5 and SKD1 and Its Role in Endosomal Trafficking to the Vacuole in Arabidopsis
Source: Front Plant Sci. 2017 Nov 20;8:1969. doi: 10.3389/fpls.2017.01969 (PMC5701936; doi:10.3389/fpls.2017.01969)
Supplement: Supplementary file 2 [file Data_Sheet_1.PDF]

Figure S1

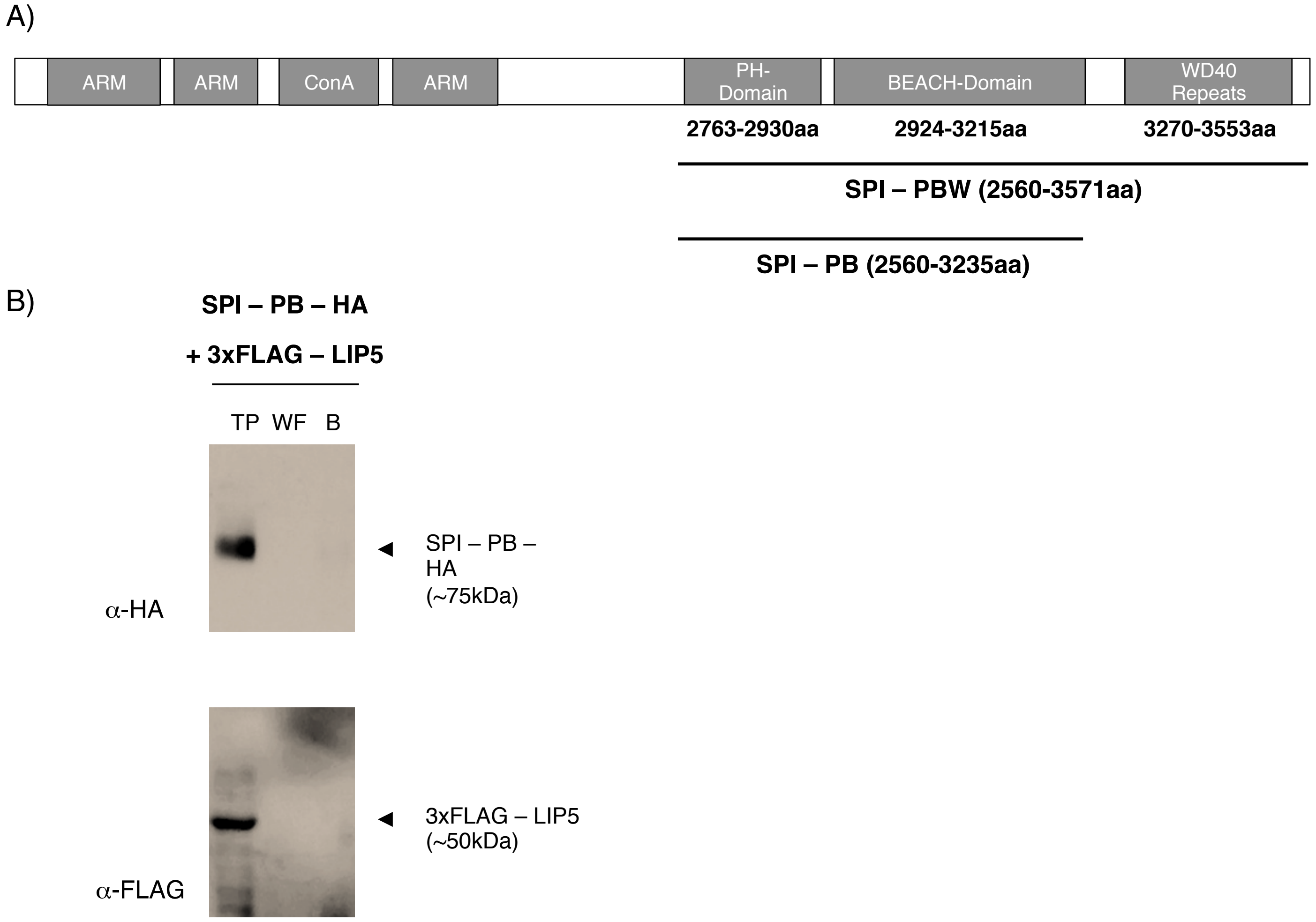

Figure S1. Domain structure of SPI and control experiments (related to Figure 1).
A) Schematic diagram illustrating the domain structure of the SPI cDNA and the relative positions of truncated constructs used in this study. ARMADILLO repeats (ARM), Concanavalin A-like lectin domain (ConA), the C-terminal PH-BEACH-WD40 module (SPI-PBW) and the PH-BEACH domains (SPI-PB) comprising fragment are presented. B) Negative control for co-IP assays. α-ProteinA conjugated beads were incubated with lysates of transfected *N. benthamiana* leaves expressing 3xFLAG-LIP5 or co-expressing SPI-PB-HA and 3xFLAG-LIP5. Throughput (TP), the last wash fraction (WF) and the bead fraction (B) are presented. No unspecific binding to α-ProteinA beads could be detected by α-HA or α-FLAG antibody staining on Western blots. Arrowheads indicate expected protein sizes.

Figure S2

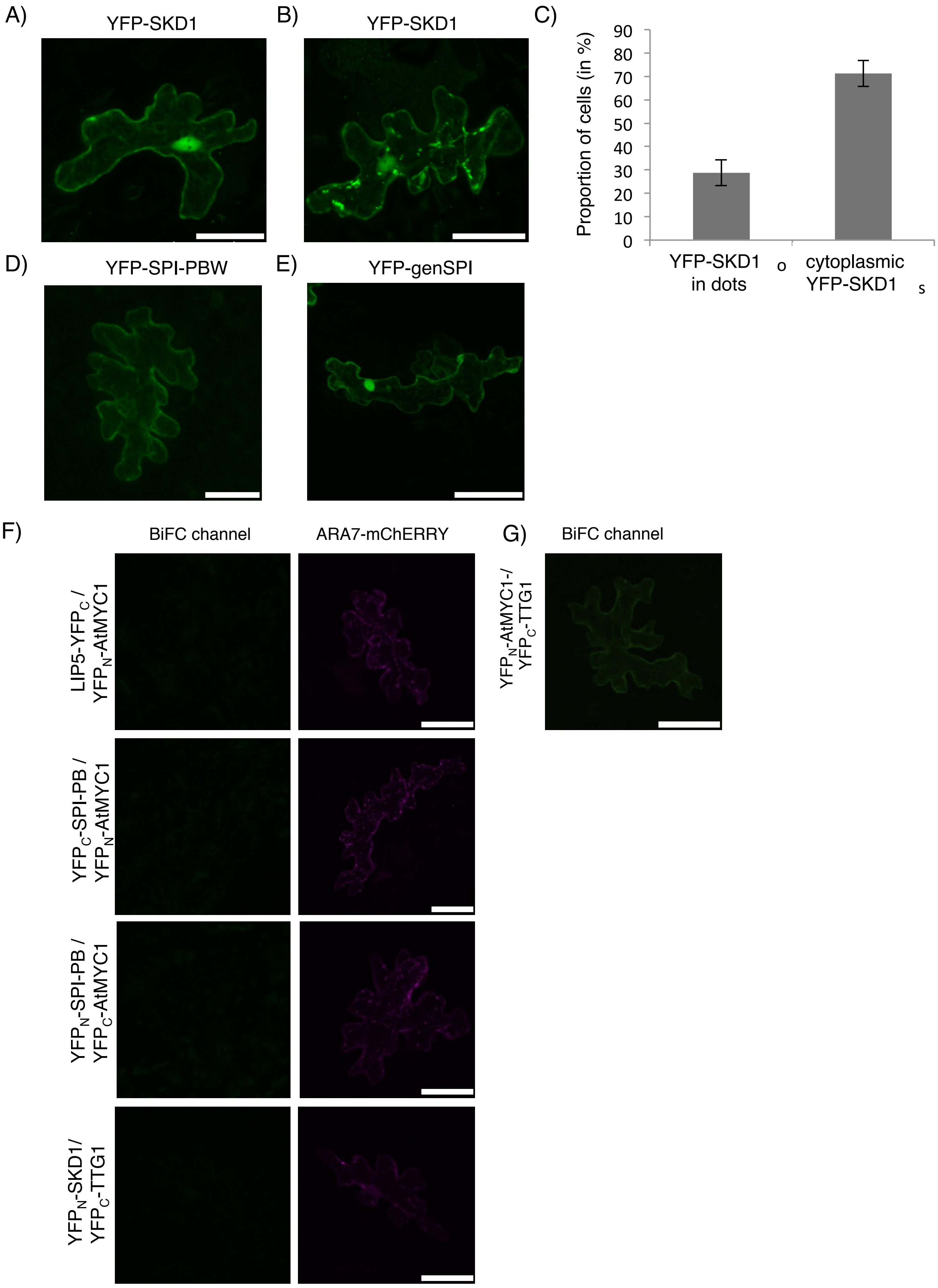

A)
YFP-SKD1
B)
YFP-SKD1
C)
Proportion of cells (in %)
90
80
70
60
50
40
30
20
10
0
YFP-SKD1 in dots
cytoplasmic YFP-SKD1
D)
YFP-SPI-PBW
E)
YFP-genSPI
F)
BiFC channel
ARA7-mChERRY
LIP5-YFPC / YFPN-AtMYC1
YFPC-SPI-PB / YFPN-AtMYC1
YFPN-SPI-PB / YFPC-AtMYC1
YFPN-SKD1/ YFPC-TTG1
G)
BiFC channel
YFPN-AtMYC1-/ YFPC-TTG1

Figure S2. Analysis of proteins *in planta* and BiFC negative controls (related to Figures 2 and 3). Transiently transformed Arabidopsis leaf epidermis cells showing (A) cytoplasmic YFP-SKD1 and (B) dot-localized YFP-SKD1. Scale bars: 50μm. C) Proportion of cells showing cytoplasmic and dot-localized YFP-SKD1 (in %). Data represent the average of three biological replicates (n>30 cells each). Error bars represent SDs. D) Cytoplasmic distribution of YFP-SPI-PBW and E) YFP-genSPI in transiently transfected Arabidopsis leaf epidermis cells. Scale bars: 50μm. F) Representative images of cells expressing BiFC negative controls in combination with mCHERRY-ARA7 as transformation control. SPI-PB, N-terminally fused to $YFP_{N/C}$-fragments, and LIP5-YFP-$_C$ were co-expressed with AtMYC1, N-terminally fused to the corresponding YFP-fragments. YFP_N-SKD1 was co-expressed with YFP_C-TTG1. Scale bars: 50μm. G) Representative image of BiFC-signal in cells co-expressing YFP_N-AtMYC1 and YFP_C-TTG1. Scale bars: 50μm.

Figure S3

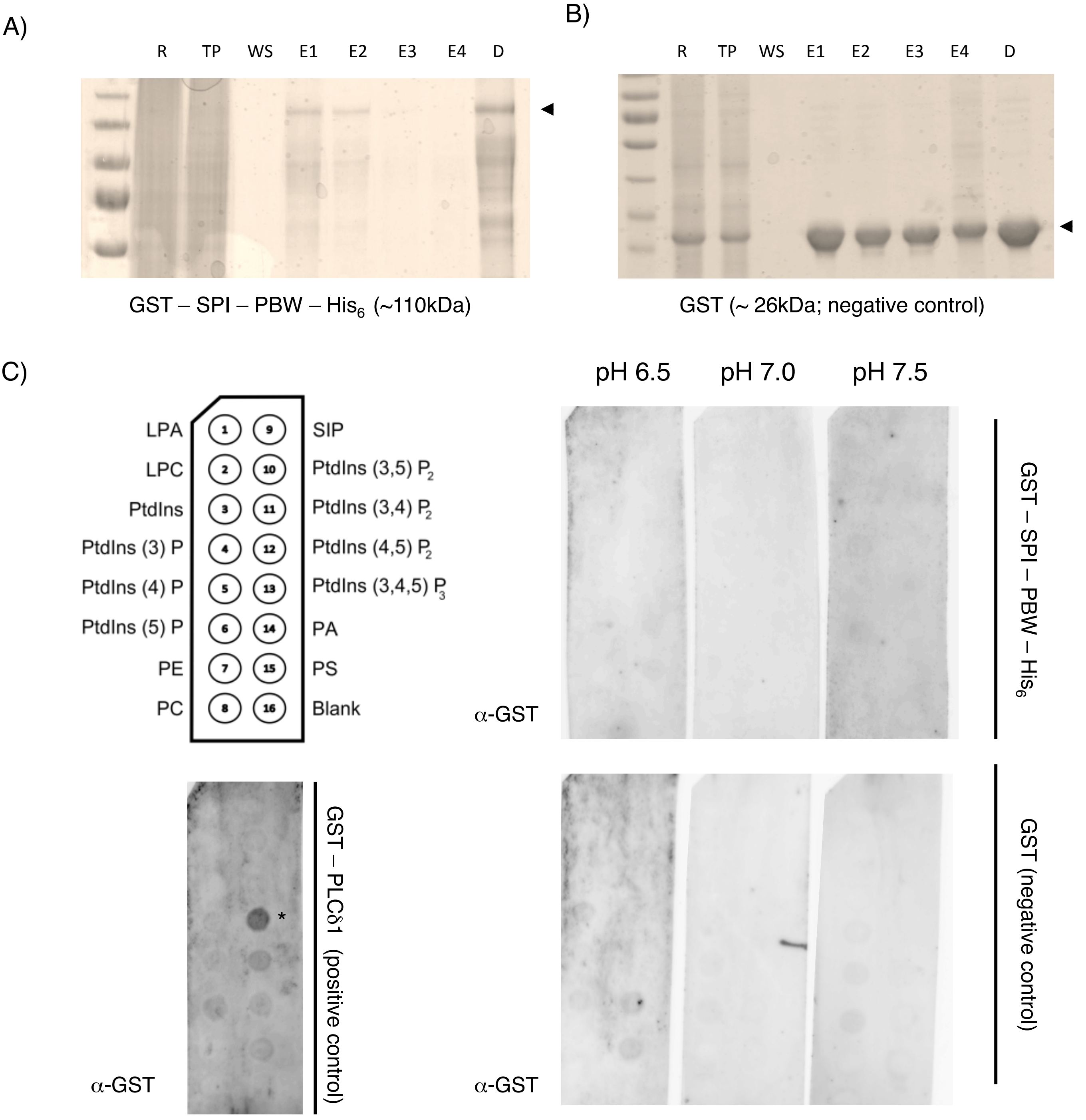

Figure S3. Protein purifications and protein-lipid binding assay (related to Figure 3).
Purifications of A) GST-SPI-PBW-His_6 and B) GST presented on Coomassie-stained gels. Arrowheads indicate expected protein sizes. C) Protein-lipid binding assays performed at pH 6.5, pH 7.0 and pH 7.5 in TRIS buffer (100mM TRIS; 150mM NaCl). Diagram represents arrangement of immobilized lipids (100pmol) spotted on nitrocellulose membranes. α-GST antibody staining demonstrated that GST-SPI-PBW-His_6 and GST negative control did not bind to any phospholipid tested. Assay-functionality was shown by including the GST-tagged PLC-d1 PH domain protein as positive control that was clearly bound to PI4.5P (indicated by *).

Figure S4

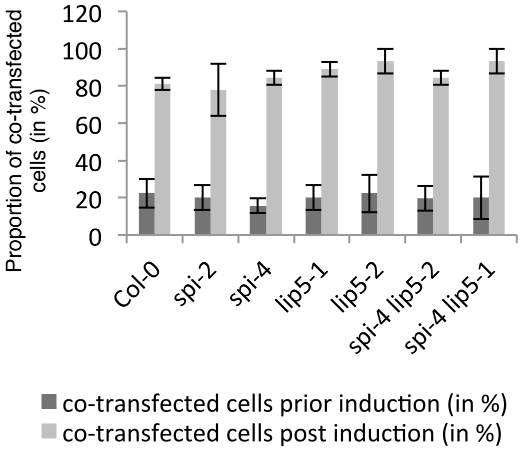

Figure S4. Analysis of EtOH-inducible constructs in transiently transformed leaf epidermis cells (related to Figure 5).

Proportion of cells showing a signal for alcR_{pro}:CPY-mCHERRY before and after induction of protein expression (in %). Data represent the average from three biological replicates (n=15 cells each).
